# Acetate of Lead in Cholera

**Published:** 1834

**Authors:** 


### IV.—Acetate of Lead in Cholera.

*Note on the efficacy of the Acetate of Lead, in several cases of Epidemic Cholera, in Cincinnati, October,* 1834.—By the Editor.

In various numbers of this Journal, we have given some account of the different invasions of the Cholera, which this city has experienced since the 30th day of September, 1832. In an article, the first of our 30th number, we stated, that on the 18th of September, the city was healthy, though a death now and then occurred from the lingering influence of the epidemic poison. For the ensuing three weeks, this influence was felt less and less every day, so that by the 7th of October, it appeared to have near or quite vanished. On the 9th and 10th there was rain, followed on the night of the latter, by a great depression of the temperature, and *immediately the epidemic was revived*, with a malignity equal to any it had previously displayed. It was not, however, general throughout the city; at least in the same degree, and many parts scarcely felt its influence. What appeared the most remarkable, was, that Fourth Street, running, (as we may inform the distant reader,) from East to West, on the upper of the two plains on which the city is built, at an average distance of four squares, or more than a third of a mile, from the river, was far more affected than any other. Of all the streets of the city, this is well known to be one of the cleanest, and the most entirely devoted to private dwellings; which are, with few exceptions, not crowded, and generally of an elegant and commodious character. Along this street, or within half a square of it, there occurred, on the 11th, 12th and 13th of the month, no less than nine deaths; although its population is far from being dense. Had an equal, proportionate mortality, prevailed over the whole city, it would have lost, in those three days, at least 250, instead of 25, which was about the actual number. The duration of this outbreak was short, and before the end of the month, it was rare to meet with a single case. Those who fell victims were, generally, persons in comfortable or affluent circumstances, of both sexes, and at all periods of life, from childhood to old age.

Now, what shall we say of these facts? That Epidemic Cholera depends on changes in the sensible qualities of the atmosphere? Certainly not—for such a change as that which occurred on the 10th of October, has taken place a countless number of times, without producing Cholera. It was, in truth, but a powerfully exciting cause, increasing the susceptibility of the system, and impairing the functions of the skin, before the remote or specific cause, had left our atmosphere, or while the systems of some, were still, in the inappreciable condition, which extends from the impress of the specific poison, to the manifest developement of morbid actions. Had the great change of weather been postponed, perhaps a fortnight, its power of reproducing the epidemic might not have been perceptible. It is by neglecting these obvious views, that many physicians, have been led into the untenable conclusion, that Epidemic Cholera arises,*ab initio*,from atmospheric vicissitudes.

We may mention two facts which seem to indicate, that mere depression of temperature, is not the sole exciting cause, in cases of this kind; but that in changes of weather, there are other conditions of the atmosphere, than those of which our senses take cognizance, on which the pernicious effects, in part, at least, depend. Two of the victims to which we have referred, were females confined to the house. One a lady, in middle life, who had been ill of bilious fever, and was not yet able to go abroad; the other, an elderly woman, whose age and infirmities detained her in the chamber; neither of whom, could be fairly, considered as under the influence of external changes of temperature.

We may also state, that the cause which awakened the Epidemic, although extensive in its geographical range, was powerless elsewhere than in Cincinnati, apparently, because the specific influence was not in being. Two cases occurred, which would seem to be exceptions to this remark; but they were not. Professor McGuffy, of Oxford, where the disease had recently prevailed, and Thomas S. Grimke, of South Carolina, who had spent nearly three weeks in Ohio, and the last two in Cincinnati, left our city in good health, on the

10th,—the day on which the change of weather took place. The former sailed to Louisville and the latter travelled towards Columbus. They were both attacked, the latter in the course of the night,—and the former in two days; the people in the regions—300 miles apart—into which they went, being at the time free from the Epidemic. As a few persons still cherish the opinion, that Cholera, is contagious, we may add, that although Mr. Grimke's attack proved fatal, the disease did not spread among those who surrounded him.

But the chief object of this little paper is, to state the results of some experience with the Acetate of Lead, in several cases of our October invasion.

A short time before it occurred, being told by a medical friend, that a physician of Canada had lately used that medicine in large doses,with advantage, in the treatment of Cholera, we determined, when opportunity should offer, to give it a trial.

The first case in which we used it was that of Professor McGuffy, mentioned above. He returned from Louisville on the 15th, labouring under a *serous* diarrhœa, which had commenced on the 11th or 12th. His strength was much reduced. He was put to bed, and took calomel and opium with the cretaceous mixture. For several hours, through the middle of the day, the disease seemed suspended, but towards evening it returned, with profuse water gruel discharges, accompanied with nausea and a rapid sinking of the pulse. As vomiting and spasms were impending, we resolved on the immediate administration of the Acetate, in liberal doses; and accordingly gave a pill of ten grains, with one of opium, every hour, till he took half a drachm. The diarrhœa ceased after the second dose; but he continued with a strong disposition to vomit, notwithstanding the application of a sinapism to the epigastrium. Soon after the third dose, he said his stomach felt like the skin where the mustard had been applied, and his pulse became excited. He was then made to take a draught of salt and water, to decompose the Acetate, and was soon relieved from the gastric irritation. The night was one of considerable febrile excitement. The suspended alvine evacuations, were restored the next day, with healthy secretions from the liver, and he was soon well enough to return to Oxford.

On the same day in which Prof. McGuffy was ill, a little boy, 10 years old, was still worse with the same malady. He was taken in the night, and we did not see him till the following afternoon. He had vomiting, and a profuse diarrhœa; the discharges resembling milk and water. His pulse had failed very much. A single powder of ten grains of the Acetate of Lead and one of opium, suspended every symptom, and the next morning he was able to sit up. Without the use of calomel, his discharges assumed a bilious character.

About the same period, six or eight persons, male and female, affected with the copious watery diarrhœa, which manifests the epidemic influence, took the same compound. In every instance, it arrested the disorder, and without the use of calomel, there followed bilious evacuations.

At a period somewhat subsequent, a blind pauper arrived in the city, from the Upper Mississippi, and applied to us to be operated on for cataract. Although no Cholera existed here at that time, he was soon taken down. Being upwards of 80 years of age, (though a robust old man,) his case was every way threatening; especially as vomiting and spasms, attended with profuse rice water discharges, were present, and had existed for some time, when we were called to prescribe.—An exclusive reliance was placed on the Acetate of Lead and opium, given in repeated doses of five grains of one and half a grain of the other, and under this treatment he recovered—the functions of the liver being restored without the use of mercurials.

In one case of collapse, a patient of Dr. Shotwell's, we saw the same medicine administered without effect.

In no instance, did any sinister effects result from the doses that have been mentioned; and on no person, but Prof. McGuffy, who took 30 grains in two hours, did they produce any sensible effect of an unpleasant kind. Should the dose, in any instance prove too strong, it may be promptly decomposed and rendered inert, by the alkaline muriates or sulphates.

We deem it unnecessary to extend this loose and desultory report, the sole object of which is to call the attention of our brethren, who, in the common course of things, have yet to grapple with this pestilence, to this medicine, which we earnestly hope they will submit to the test of experience. Original and constant prescribers of Calomel, for two years, we have often witnessed its anticholeric power; but have often seen it fail. The total cessation of the functions of the liver has seemed to require it, and theoretical views not less than experience have contributed to its extensive administration. We are entirely satisfied, however, that the secretion of bile will return, if that of serum be arrested; and, of the power of the Acetate to effect this, we think some evidence has been offered. The preparation of lead, moreover, acts much more promptly, than that of mercury; and its power of exciting in the stomach the kind of inflammatory irritation, which Mr. McGuffy experienced, appears to us, to be one of its highest recommendations; for we have yet to learn *from observation*, that Cholera is a gastro-enteritis. So far, indeed, from consisting in inflammation, we are disposed to think, that in general, the occurrence of that state, in connexion with a suspension of the secretory action of the mucus membrane, will render the disease quite manageable. It may be said, however, that all kinds of stimulants have been profusely employed, and must often have excited gastritis; this we grant; but were they fitted to restrain the inordinate action of the intestinal mucous membrane? We think not, and their failure may have arisen from that very imperfection. The great question in this matter may be thus stated—Is the failure in the hepatic secretion the cause, or the effect, of the profuse secretion from the bowels? Most of the prescriptions in Cholera have been based on the first alternative, in this interrogatoty; but how often do we see the actions of the liver suspended, without the least increased secretion from the bowels? and how consonant is it with many well ascertained pathological, indeed physiological phenomena, for a great increase in one secretion, to be followed by a failure or total suspension of some other;—in Cholera, it would seem of several others, for those of the skin, and kidneys, are as much reduced, as that of the liver. If then the increase of the muco-serous secretion from the intestinal ramifications of the portal circle, precedes and occasions the diminished secretion from the hepatic extremities of that system, why should we not pass by the latter and direct our remedies upon the former? Striking at the root, instead of the branches of the tree.

It may be said, that most of the cases, from which have drawn a conclusion favorable to the powers of the Acetate of lead, were not so far advanced as to justify the inference, that it is a remedy for Cholera. But what *can* cure that disease, when vomiting and spasms, with reduced calorification and cutaneous secretion, are fully established? According to the books (which have fallen on the profession in epidemic profusion,) at least a thousand medicines! to which cures, if collected into one great volume, might be applied the zoological maxim, that all *numerous progenies are vicious.* We believe, that each has been occasionally successful, but generally ineffectual. For the hundredth time we here repeat, as the result of our own observation, and that of the profession in Cincinnati, that before the pulse has sunk very low and the muscular system been seized with spasms, the disease is more tractable than hysteria; after those symptoms have become established, more *incurable* than apoplexy.

That its advancement is, *generally*, slow enough to admit of the application of appropriate remedies, if the people would do their duty to themselves and the profession, we know from abundant observation. If they will not, they should not hold their medical friends responsible. The man who dies of small pox, from neglecting vaccination, and he who loses his life from indifference to the curable stages of Cholera, perish from different violations of the same law of self-preservation—deferring the appropriate means, till their application can be of no avail. It was by violating this law, that the distinguished citizen of South Carolina, whom we have just mentioned, was lost to his family and the country. As many of our readers are familiar with his reputation and fate, we shall close this article with a brief notice of his last illness and death.

### Thomas S. Grimke.

Mr. Grimke came from Charleston to this city, for the purpose of delivering, by request, the annual oration of the Erodelphian Society, in Miami University, at Oxford, forty miles from Cincinnati. On returning, he spent two weeks in the city, and departed, apparently in good health, for Columbus, in the northern mail stage, at 2 o'clock, P. M. of the 10th of October. The night became cold, and as every hour carried him further north and upon a higher level, the depression of temperature must have been extremely pernicious to his southern constitution. At Waynesville, 42 miles from the city, the fatal cholerine had begun, and there he should have left the stage, and resorted to medical advice. But he kept on, passing through Xenia and Springfield. 10 and 30 miles further, without seeking the assistance of the profession. He encountered the high, cold and unsettled summit level between the vallies of the Miami and Scioto; and after a ride of 20 additional miles, without the use of medicines, he arrived at Anderson's tavern, (Gwynne's farm,) 24 miles west of Columbus, in a state of incipient collapse. Still bent on reaching his place of destination, he attempted, after having left one coach to enter another, but was unequal to the effort; and then did, what should have been done at Waynesville, 18 hours before. stop and lie by. The hospitable family applied heat and frictions to the surface of his body, and urged him to take calomel and laudanum; but he declined, observing that he would not take medicine without the advice of a physician. When Dr. Thompson, of Columbus arrived, he was pulseless, and expired in a few hours. Now if Mr. Grimke had received a wound at Waynesville, which led to hæmorrhage, he would have had the vessel taken up—if not, he must have bled to death; but not more certainly, than if he suffered the cholerine, which constitutes the first stage of the Epidemic, to continue unchecked—by rest, a recumbent posture and the appropriate remedies. His remains were committed to *permanent* sepulture in Columbus.

Thus perished, from mere inattention to the means of self-preservation, in the meridian of life, a most learned, benevolent, pious and useful man—whose heart embraced the Union and all its most cherished interests—and whose name and amiable fame are the property of the nation.

*December*, 1834.

